# Altered Global Signal Topography in Major Depressive Disorder With and Without Anxiety

**DOI:** 10.1155/da/3864020

**Published:** 2025-07-02

**Authors:** Huaijin Gao, Rui Qian, Wen Zhu, Yihan Ma, Dan Wu, Zhiyong Zhao

**Affiliations:** ^1^Department of Biomedical Engineering, Children's Hospital, Zhejiang University School of Medicine, National Clinical Research Center for Child Health, Zhejiang University, Hangzhou, Zhejiang, China; ^2^Department of Biomedical Engineering, College of Biomedical Engineering and Instrument Science, Zhejiang University, Hangzhou, Zhejiang, China

**Keywords:** anxiety, fMRI, global signal, major depressive disorder

## Abstract

**Background:** Major depressive disorder (MDD) is a psychiatric disorder characterized by alterations in global signal (GS) topography across various neural networks and brain regions, including the default mode network and sensorimotor-related areas. While previous research has demonstrated the potential of global brain activity measures to differentiate MDD from healthy controls (HCs), specific changes in GS distribution among MDD patients with and without anxiety remain poorly understood. This study aims to investigate anxiety-related alterations in GS topography in MDD and their associations with clinical symptoms.

**Methods:** Resting-state functional magnetic resonance imaging (fMRI) and T1-weighted imaging data were collected from 334 MDD patients with anxiety, 145 MDD patients without anxiety, and 307 HCs as part of the REST-meta-MDD consortium. We computed GS topography using GS correlation (GSCORR) and assessed structural–functional interaction (SFI) by examining the relationship between gray matter volume and GS for each subject.

**Results:** Our analysis revealed no significant differences in GS topography among the three groups at either the whole-brain or network levels. However, decreased GSCORR was observed in the right precentral gyrus, insula, and posterior parieto-occipital cortex in anxious MDD patients compared to HC. SFI analyses indicated anxiety-related alterations in the sensorimotor network, precuneus, putamen, and middle temporal gyrus. Moreover, GSCORR in the inferior parietal lobe and cerebellum exhibited specific correlation trends with anxiety and depression symptoms, respectively.

**Conclusions:** These findings underscore an abnormal topographic shift in global brain activity in MDD patients with anxiety, offering a new insight into understanding brain dysfunction associated with this disorder.

## 1. Introduction

Major depressive disorder (MDD) is a severe mental illness that imposes a substantial burden on society. In China, ~56 million individuals are affected by depressive disorders, with a prevalence rate of 3.1% [1]. Notably, 75.9% of these individuals experience varying degrees of functional disability [2]. Depression often coexists with other conditions, particularly anxiety, which is one of the most common comorbidities [3]. Approximately 45%–67% of MDD patients meet the criteria for at least one comorbid anxiety disorder [4]. While comorbid anxiety disorders refer to individuals who meet diagnostic criteria for both MDD and an anxiety disorder, the DSM-5 introduced the ‘anxious distress' specifier, characterized by symptoms such as tension, restlessness, difficulty concentrating, and fear of something awful happening or losing control [5], concurrent anxious symptomatology refers to individuals exhibiting anxiety symptoms that do not necessarily meet the threshold for a formal anxiety disorder diagnosis. The dimensional diagnosis of anxious depression typically involves a diagnosis of MDD (based on either DSM or ICD criteria) alongside anxiety symptoms (based on cutoff scores from standardized scales, such as HAMA) [6].

While prior studies have identified distinct neurobiological and clinical features between MDD patients with and without anxiety symptoms [7, 8], the neurobiological mechanisms underlying anxiety following depression remain unclear. Resting-state functional magnetic resonance imaging (fMRI) studies have documented regional functional disruptions in MDD patients with anxiety, using conventional measures such as amplitude of low-frequency fluctuations (ALFF) and fractional ALFF (fALFF) [9–12]. The temporal gyrus plays a critical role in auditory processing and language comprehension [13]. Research indicates that the bilateral superior temporal gyrus and left middle temporal gyrus are key regions associated with nonanxious depression, while the right superior temporal gyrus plays a significant role in the neural basis of anxious depression [10, 11]. Additionally, anxious depression has been linked to ALFF alterations in the anterior insula, which is essential for internal sensory processing [14, 15], and the anterior cingulate cortex, which plays a crucial role in emotional regulation [9, 10, 16]. Individuals with anxious depression also show reduced ALFF in the right orbital part of the middle frontal gyrus [12] and increased ALFF in the right dorsal anterior insular cortex and bilateral lingual gyrus [10] compared to those with nonanxious depression. Despite these findings, studies examining alterations in the topographical patterns of whole-brain activity are limited. The human brain regions operate in a coordinated manner rather than independently [17, 18]. Research suggests that the overall topographical pattern and interactions among brain networks correlate with anxiety symptoms [19–21]. For instance, higher global brain connectivity in the ventromedial prefrontal cortex and precuneus is linked to lower trait anxiety [19], while social anxiety disorder has been shown to lead to abnormal topography in the default mode and salience networks [20]. These findings imply that whole-brain topographical activity patterns, which may reflect the dominance of certain behavioral functions over others at a psychopathological level [21], may play a critical role in the neural mechanisms underlying anxiety disorders.

The global signal (GS) represents the averaged signal of gray matter (GM) voxels and reflects the overall fluctuation of global blood oxygen level-dependent (BOLD) activity [22]. The role of GS has been debated in fMRI studies; it has traditionally been considered a non-neuronal signal resulting from artifacts such as head motion, hardware, respiratory factors, and other unknown effects [23, 24] and is typically regressed out during data preprocessing. However, recent studies suggest that GS may have a neural basis [25, 26] and is associated with conscious states [27], mental states [28], human cognition [25, 29], and clinical conditions such as epilepsy [30] and schizophrenia [31, 32]. GS topography, which captures the spatial distribution of GS activity through global signal correlation (GSCORR), provides a novel approach to explore the relationship between global and local neural functioning [23]. Studies have shown that MDD patients exhibit abnormal GS topography, shifting from unimodal lower-order sensory and motor regions to transmodal higher-order associative regions like the default mode network (DMN) [18]. The DMN, comprising the medial prefrontal cortex, posterior cingulate cortex, and angular gyrus, is primarily active during self-referential thinking and emotional processing [33]. In patients with anxiety–depression, DMN activity has been associated with excessive rumination and impaired perceptual processing [34]. Studies have also reported reduced static GS topography in the bilateral parahippocampal and hippocampal gyri, as well as increased dynamic GS topography variability in the right ventromedial prefrontal cortex in MDD patients [35]. Furthermore, MDD patients exhibit increased GS contributions in highly interconnected hubs and reduced GS influence in frontoparietal and occipitotemporal regions compared to healthy controls (HCs). These alterations are associated with deficits in cognitive control and visual attention [36, 37]. Scalabrini et al. [38] discovered increased GSCORR in DMN in MDD patients and used GSCORR to accurately classify MDD patients versus healthy subjects. These findings suggest that alterations in GS topography are associated with depression and may serve as a biomarker for identifying subtle disruptions in brain function. Understanding the characteristics of GSCORR is essential for comprehending the neural mechanisms underlying anxiety in MDD. However, studies examining GS alterations in MDD patients with and without anxiety symptoms are scarce.

In this study, we utilized the Chinese REST-meta-MDD database [39] to explore: (1) changes in GS topography between MDD patients with and without anxiety at the whole brain, network, and regional levels; (2) the underlying structural mechanisms of GSCORR alterations in MDD; and (3) the associations between GS topography alterations and clinical assessments. We hypothesized that patients with anxious depression would exhibit more pronounced reductions in GS topography compared to those without anxiety and that GSCORR might reveal different brain abnormalities than ALFF or fALFF.

## 2. Methods

### 2.1. Participants

**Figure 1:**
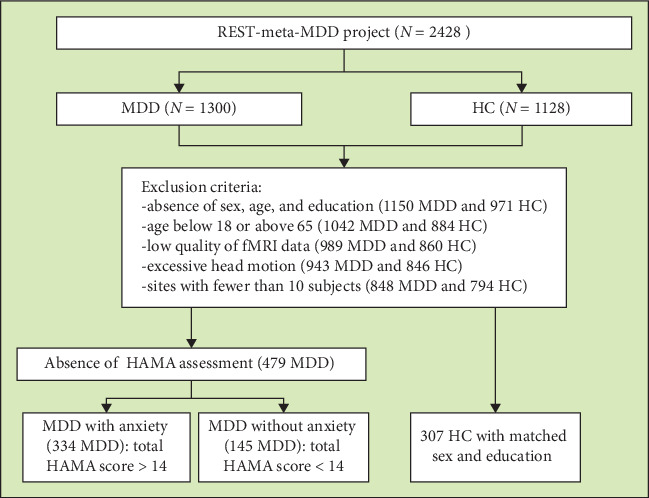
Flowchart of the study. MDD: major depressive disorder; HC: healthy control; HAMA: Hamilton Anxiety Rating scale.

We screened resting-state fMRI and structural T1-weighted MRI images of MDD patients with anxiety (*N* = 334), those without anxiety (*N* = 145), and HCs (HC, *N* = 307) from the Chinese REST-meta-MDD database [39]. The exclusion criteria were as follows: (1) lack of information on sex, age, and education; (2) age below 18 or above 65 years; (3) poor imaging quality or bad spatial normalization, assessed by visual inspection; (4) excessive head motion (average framewise displacement >0.2 mm) or inadequate coverage (<90% of the group mask); (5) spatial correlation <0.6 (a threshold defined as mean - 2SD) between each participant's regional homogeneity (ReHo) map and the group mean ReHo map; (6) absence of Hamilton Anxiety Rating Scale (HAMA) assessment; and (7) sites with fewer than 10 subjects. A detailed flowchart is shown in Figure 1. A diagnosis of MDD with anxiety was established for patients with a total HAMA score exceeding 14 [9]. No specific cut-off score for the Hamilton Depression Rating Scale (HAMD) was applied in our study. The HAMD was used to assess symptoms of depression.

All study sites obtained approval from their local institutional review boards and ethics committees, and all participants provided written informed consent.

### 2.2. Data Preprocessing

Structural T1-weighted MRI and resting-state fMRI images were preprocessed at each site using standardized DPARSF (http://rfmri.org/DPARSF) processing parameters [40]. Initially, T1-weighted images were segmented into three tissue classes: GM, white matter, and cerebrospinal fluid [41]. Next, transformations were computed to map each participant's images from native space to Montreal Neurological Institute (MNI) space using the Diffeomorphic Anatomical Registration Through Exponentiated Lie algebra (DARTEL) tool [42]. These transformations were then applied to the GM concentration maps. To generate normalized gray matter volume (GMV) maps, the normalized GM concentration maps were multiplied by the nonlinear determinants produced during normalization. The final GMV maps were resliced to a voxel size of 1.5 mm^3^ and smoothed using an 8 mm full-width at half-maximum Gaussian kernel.

For fMRI data, we discarded the first five functional volumes and processed the remaining volumes for slice-timing and head-motion corrections. We regressed out the white matter, cerebrospinal fluid, and the Friston 24-parameter head motion model to account for nuisance variables. The data were then spatially normalized to an EPI template aligned with MNI space and transformed into the frequency domain using fast Fourier transformation. The average square root of the power spectrum within the 0.01–0.1 Hz range was calculated as the ALFF value. The fALFF was derived as the ratio of the low-frequency power spectrum (0.01–0.1 Hz) to the entire frequency range [40]. All ALFF/fALFF maps were *Z*-standardized by subtracting the mean value of the entire brain from each voxel and dividing by the corresponding standard deviation, followed by smoothing (6 mm FWHM). GS was extracted for each subject by averaging the time series of voxels within a whole-brain GM mask. Then, we used the DOS-160 atlas [43] to segment the brain into 160 regions of interest (ROIs) involved in six networks: cingulo-opercular network (CON), fronto-parietal network (FPN), DMN, sensorimotor network (SEN), occipital network (ON), and cerebellar network (CEN). For each ROI, the GSCORR was calculated as the GS topography using the Pearson correlation coefficient [40, 44], followed by Fisher's r-to-z transformation. Additionally, the product of GSCORR and GMV for each ROI was defined as structural–functional interaction (SFI) [45].

### 2.3. Statistical Analysis

To control for potential confounding factors, we employed a linear mixed effects (LMEs) model for our statistical analyses. The LME model describes the relationship between a response variable and independent variables (here, diagnosis and covariates such as age, sex, education, head motion, and total GMV). To account for the potential confounding effects from different sites, we treated site as a random effect in the model, which accounts for the variability between sites while focusing on the fixed effects of interest [39, 46, 47]. The coefficients can vary for grouping variables (here, site). We utilized MATLAB's command fitlme (https://www.mathworks.com/help/stats/fitlme. html) to test the model: y ∼1 + Diagnosis + Age + Sex + Education + Motion + total GMV + (1|Site) + (Diagnosis | Site). This approach provides t and *p* values for the fixed effect of Diagnosis [39]. We conducted across-group comparisons of all measures to evaluate the differences between groups at whole brain, network, and regional levels, respectively. Multiple comparisons were corrected using the false discovery rate (FDR) method (*p*  < 0.05) for each measure. The highest correction factor was based on the total number of comparisons at each level (whole brain, network, and regional). Different measures (e.g., GSCORR, SFI) were considered separate families of comparisons, while different levels (e.g., whole brain, network, regional) were treated as a single family of comparisons. Then, we evaluated correlations between GSCORR and clinical assessments in each patient group using LME, replacing the response variable with total 17-item HAMD scores or HAMA scores, respectively.

## 3. Results

### 3.1. Demographic Characteristics

There were no significant differences in gender, education, and head motion during MRI scan among the three groups but showed a significant difference in age. MDD patients with anxiety had a longer duration of illness, higher HAMD and HAMA scores compared with those without anxiety (Table 1).

### 3.2. The GSCORR Differences Between Groups

**Figure 2:**
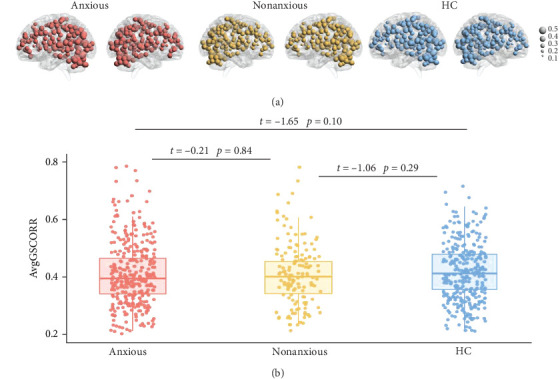
(A) Average GSCORR map for each group. The size of the node represents the average value of GSCORR in each ROI. (B) GSCORR differences between groups at the whole brain level. The positive and negative t values represent larger and smaller GSCORR values in the former group than latter group, respectively. No significant difference in GSCORR was observed at the whole-brain level. Anxious: major depressive disorder patients with anxiety; nonanxious: major depressive disorder patients without anxiety; HC: healthy control; AvgGSCORR: average value of GSCORR.

**Figure 3:**
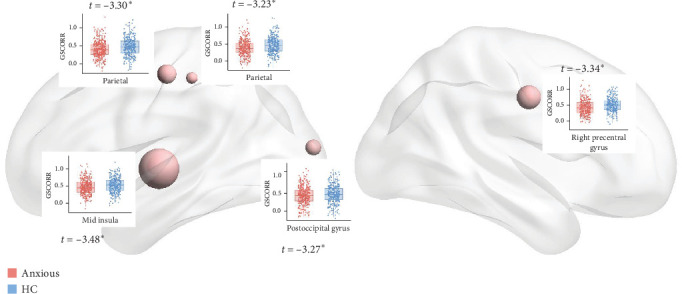
GSCORR differences between anxious MDD and HC at the regional level. The regions that survived after FDR correction are shown. The size of the nodes represents the t-value derived from LME.*⁣*^*∗*^: 0.01 < *p*  < 0.05.

Figure 2 presents the averaged GSCORR map for each group. No significant difference in GSCORR was observed at the whole brain and network levels. At the regional level (Figure 3), compared with HC, anxious MDD showed decreased GSCORR in the right precentral gyrus, insula, and posterior parieto-occipital cortex, which were not observed in nonanxious MDD. No significant difference in GSCORR was observed at the regional level when comparing nonanxious MDD patients to anxious MDD or HC.

### 3.3. The SFI Differences Between Groups

**Figure 4:**
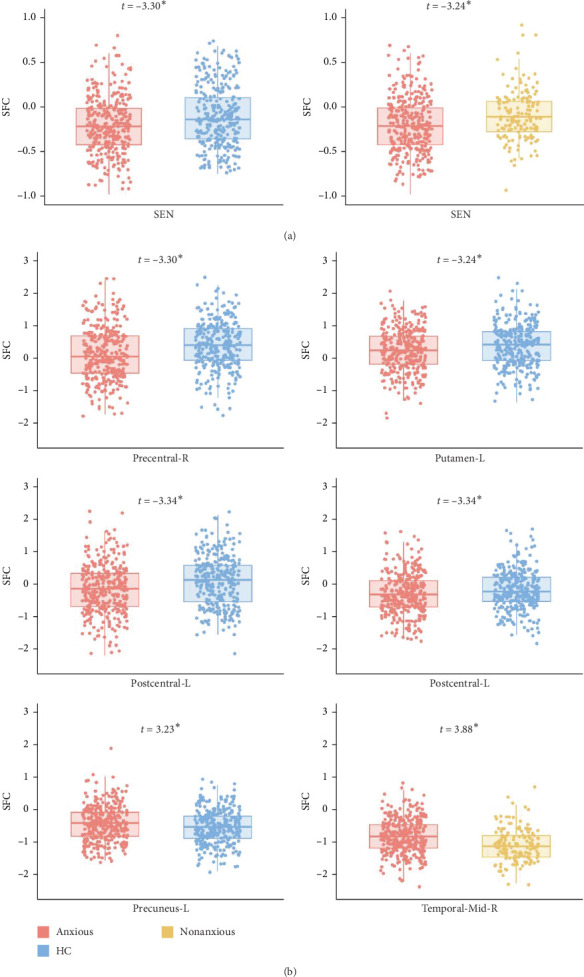
Structural–functional interaction differences between groups at the network level (A) and at the regional level (B). SFI: structural–functional interaction; SEN: sensorimotor network. *⁣*^*∗*^: 0.01 < *p*  < 0.05.

The LME did not reveal any significant differences in SFI at the whole-brain level. However, at the network level, anxious MDD patients exhibited decreased SFI in the SEN compared to both nonanxious MDD patients and HC (Figure 4A). At the regional level, anxious MDD showed decreased SFI in the right precentral gyrus, left postcentral gyrus, and left putamen, along with increased SFI in the left precuneus when compared to HC. Furthermore, increased SFI in the right middle temporal gyrus was observed in anxious MDD patients compared to nonanxious MDD (Figure 4B). No significant difference in SFI was observed at both whole brain and network levels when comparing nonanxious MDD patients and HC.

To determine whether the alterations in SFI were influenced by structural changes, we compared GMV differences between groups at the whole brain, network, and regional levels (Figure S1). Nonanxious MDD patients showed decreased GMV in the frontoparietal and ONs and increased GMV in the left precuneus compared to HC. In contrast, anxious MDD patients exhibited increased GMV in the right inferior frontal gyrus compared to nonanxious MDD (Figure S2). Importantly, the regions displaying significant alterations in SFI for anxious MDD did not show significant changes in GMV. Thus, the SFI alterations in anxious MDD may primarily result from functional disruptions rather than structural changes.

### 3.4. Correlation Between GSCORR and Clinical Assessments

**Figure 5:**
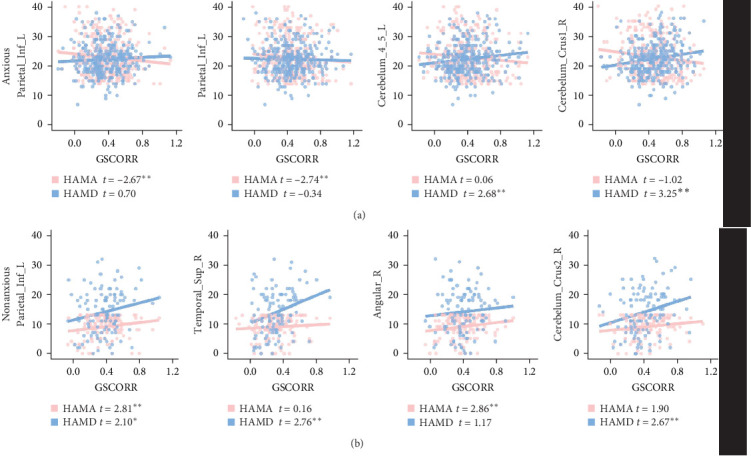
Correlation between GSCORR and clinical characteristics in anxious MDD group (A) and nonanxious MDD group (B). HAMD: Hamilton Depression Rating Scale; HAMA: Hamilton Anxiety Rating Scale. *⁣*^*∗*^: 0.01 < *p*  < 0.05; *⁣*^*∗∗*^: 0.001 < *p*  < 0.01.

In the anxious MDD group, we observed negative correlations between GSCORR and HAMA in the left inferior parietal gyrus, along with positive correlations between GSCORR and HAMD in the superior cerebellum (Figure 5A). In the nonanxious MDD group, positive correlations were found between HAMA and GSCORR in the right angular gyrus and left cuneus, as well as between HAMD and GSCORR in the inferior frontal gyrus, superior temporal gyrus, and inferior cerebellum (Figure 5B). Notably, both HAMA (*t* = 2.81, *p*=0.006) and HAMD (*t* = 2.10, *p*=0.03) exhibited positive correlations with GSCORR in the inferior parietal gyrus (Figure 5B and Figure S3). However, these correlations did not survive FDR correction (*p*  < 0.05).

### 3.5. Alterations of ALFF and fALFF in MDD With and Without Anxiety

**Figure 6:**
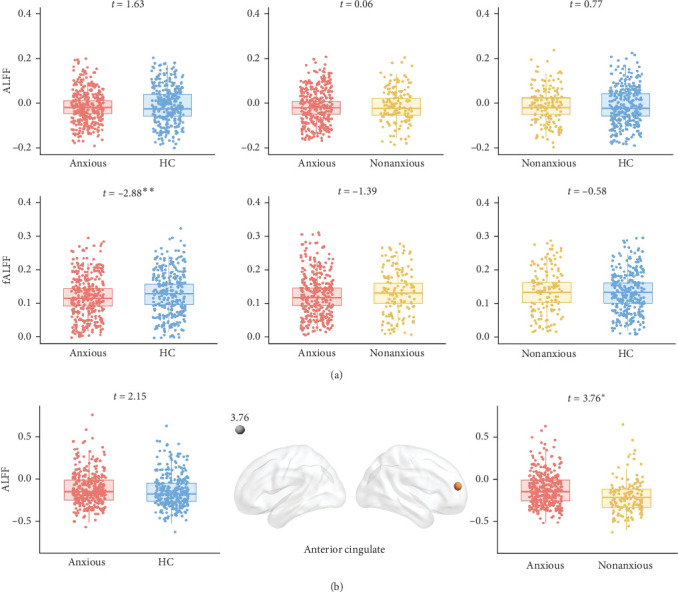
ALFF and fALFF differences between groups at the whole-brain level (A) and the regional level (B). *⁣*^*∗*^: 0.01 < *p*  < 0.05; *⁣*^*∗∗*^: 0.001 < *p*  < 0.01.

As shown in Figure 6, anxious MDD showed decreased fALFF compared with HC at the whole brain level (Figure 6A) and showed increased ALFF in the right anterior cingulate gyrus compared with nonanxious MDD at the regional level (Figure 6B). No significant differences were observed at the network level.

## 4. Discussion

In this study, we examined alterations in GS topography in MDD with and without anxiety and explored their associations with clinical assessments. Our main findings were as follows: (1) anxious MDD patients exhibited decreased GSCORR in the insula and precentral gyrus, extending to the posterior parieto-occipital cortex, compared to HC; (2) anxious MDD patients showed decreased SFI in the SEN and increased SFI in transmodal regions relative to both nonanxious MDD patients and HC; and (3) GSCORR in the inferior parietal lobe and cerebellum displayed specific correlation trends with anxiety and depression, respectively. These findings suggest that GSCORR may serve as a potential biomarker for understanding the pathophysiology of anxious depression.

### 4.1. Anxiety-Related GSCORR Decreases in MDD

The insula is a critical brain region involved in internal sensory functions and processing sensory afferent information [14, 15]. Animal studies provide direct evidence of the role of the caudal insular cortex in anxiety regulation, showing that its pharmacological inactivation increases anxiety-like behavior in rodents [48]. fMRI studies in human have revealed that abnormal connectivity between insular subregions is linked to cardiac interoception and anxiety symptoms [49], and that disrupted activation in the left dorsal mid-insula is associated with both MDD and anxiety disorders [50]. Moreover, the insula plays a crucial role in cognitive and emotional processes through interactions with prefrontal regions [14, 15]. Zhang et al. reported that weaker functional connectivity between the anterior insula and orbitofrontal cortex predicted greater MDD symptom severity [51], while Stoyanov et al. found that reduced effective connectivity from the anterior insula to the dorsolateral prefrontal cortex distinguished depressive symptoms from schizophrenia-related paranoid symptoms [52]. Our findings extend these studies by demonstrating that decreased GS topography in the insula is specifically associated with anxiety in MDD. This may suggest that anxious MDD impacts the insula beyond local functional disruptions, leading to global connectivity alterations, further supporting the insula's central role in both depression and anxiety disorders [53].

Moreover, previous studies have identified regional disruptions in the precentral gyrus, including volumetric reductions and resting-state metabolic abnormalities, in MDD patients without anxiety [54] and in anxiety patients without MDD [55, 56]. The precentral gyrus is involved in processing emotional stimuli and coordinating appropriate responses through interactions with other brain regions [57]. The prelimbic and infralimbic cortices, part of the medial prefrontal cortex, are functionally similar to the precentral gyrus and are structurally connected to it in primates [58]. Studies in rats with lesions in these cortices suggest that both regions contribute to anxiety modulation and behavioral inhibition [59]. The infralimbic cortex, in particular, plays a role in suppressing behaviors associated with aversive outcomes, underscoring its importance in emotional regulation [59]. Liu et al. [55] found that reductions in ReHo and ALFF in the bilateral precentral gyrus correlated with the severity of somatic and depressive symptoms. Similarly, we observed decreased GSCORR in the precentral gyrus of anxious MDD patients compared to HC, indicating that global topography alterations in this region may be linked to both depressive and anxious symptoms. These changes may not only result from localized functional disruptions but also reflect reduced coordination and integration of the precentral gyrus within broader brain networks.

Additionally, decreased GSCORR in anxious MDD was observed in the posterior parieto-occipital cortex, which is important for visuospatial processing [60, 61]. Prior studies have shown that over-response to negative stimuli in the visual system may be associated with negative perceptual bias in MDD [62], and altered cortical connectivity in the parieto-occipital cortex is linked to impaired attention modulation [63]. We speculate that the decreased GSCORR in posterior parieto-occipital cortex may reflect reduced coordination with other brain areas, potentially contributing to negative perceptual biases in MDD.

### 4.2. The Alterations of SFI in MDD With Anxiety

SFC is traditionally defined as the correlation between white matter pathways and functional synchrony, indicating that functional communication depends on local white matter pathways [64]. Previous studies have consistently reported higher SFC in unimodal regions and lower SFC in transmodal regions among MDD patients [65]. However, as our study focuses on GSCORR—distinct from inter-regional functional connectivity—the conventional definition of SFC cannot be applied. In linear models, interaction terms are commonly used to quantify relationships between variables [66, 67], capturing how the effect of one variable depends on the level of another [68]. Therefore, we adopted the product of regional GSCORR and GMV to characterize the SFI in this study. This multimodal MRI-based feature has been utilized in clinical research on cognitive impairment [69] and Alzheimer's disease [47]. For a given brain region, the product of GSCORR and GMV may reflect the combined functional and structural contributions of that region to whole-brain dynamics.

At the network level, decreased SFI was observed in the SEN in anxious MDD patients compared to both HC and nonanxious MDD patients. This finding aligns with previous reports of sensorimotor decoupling in MDD cohorts [70], suggesting that such uncoupling may reduce the functional distinction between association and sensory areas [71], thereby impairing sensory integration in affected individuals [72]. Although our SFI calculation method differs from that used in study [65], the observed convergence in SEN alterations may indicate a shared pathophysiology between MDD and its anxious subtype. Our regional-level results further support this, revealing significantly decreased SFI in unimodal regions, such as the precentral and postcentral gyrus. In contrast, anxious MDD patients exhibited increased SFI in transmodal regions, including the precuneus and middle temporal gyrus, which are part of the DMN associated with various cognitive activities like information evaluation, episodic memory extraction, emotion processing, and self-referential mental activity [73, 74]. This observation is consistent with previous reports of increased internetwork functional connectivity between lower-order networks and the DMN [38]. Collectively, these findings align with prior research indicating higher SFI in unimodal regions and lower SFI in transmodal regions in MDD patients [65]. Additionally, previous studies have demonstrated that GS is generally represented more strongly in sensory regions than in association areas in healthy subjects [21, 31]. MDD patients exhibit an abnormal topographic shift of global brain activity from unimodal sensory and motor regions to transmodal associative regions [18, 75]. The altered SFI observed in anxious MDD provides complementary evidence that the disorder affects brain communication patterns differently in unimodal and transmodal regions. Current investigations on SFI disruption in anxiety-related disorders are limited, with only one study reporting decreased normalized characteristic path length coupling in generalized anxiety disorder compared to HC [70].

Moreover, our analysis of GMV suggests that anxiety-related alterations in SFI may not primarily stem from structural changes. Nonanxious MDD patients showed decreased GMV in the frontal-parietal and ONs, whereas these alterations were not found in SFI. Furthermore, GMV and SFI also showed different patterns of change at the regional level. These findings indicate that the observed differences in SFI between groups may be primarily driven by functional rather than structural alterations.

### 4.3. Correlation Between GSCORR and Clinical Features in MDD Subgroup

Positive correlations between GSCORR in the cerebellum and HAMD scores were observed in both anxious and nonanxious MDD patients. Traditionally, the cerebellum is recognized for its role in motor control and coordination; however, recent studies have highlighted its significant involvement in emotion regulation, impulse control, and attention [76]. Additionally, functional abnormalities in the cerebellum are linked to depression [77, 78]. Prior research has shown increased cerebellar activity and disrupted cortical connections correlated with depression severity [79], which aligns with our findings. Furthermore, we found that the GSCORR in the inferior parietal lobe correlated with HAMA scores in both patient groups. This is consistent with earlier studies demonstrating anxiety-specific activations during cognitive tasks [80] and alterations in functional connectivity [81] in the inferior parietal lobe in anxious MDD patients.

Additionally, noninvasive brain stimulation therapies, such as transcranial direct current stimulation (tDCS), transcranial magnetic stimulation (TMS), and electroconvulsive therapy (ECT), present promising approaches for modulating GS activity in these regions and alleviating anxiety symptoms in MDD patients [82–84]. In our study, we observed a significant decrease in GSCORR in the right precentral gyrus and the insula in anxious MDD patients compared to HC. Previous studies indicate that TMS and tDCS treatments for depression can modulate brain activity in several key regions, including the right precentral gyrus, right posterior cingulate, and prefrontal cortex [28, 85]. The right precentral gyrus has been identified as a primary target for TMS-induced activation [85, 86], suggesting that TMS may help restore functional network integration by enhancing GS coordination in this region. The insula is a key hub for interoception, which is often disrupted in MDD [87] and linked to anxiety symptoms [88]. Evidence indicates that applying tDCS on the insula can modulate interoceptive awareness and may alleviate anxiety symptoms in MDD [33, 34] by modulating GSCORR in this region. Additionally, ECT improves depressive symptom in patients, which were linked to the structural and functional changes in cerebellar circuits [29, 35]. Thus, the association we observed between GSCORR in the superior cerebellum and HAMD scores, suggesting that this cerebellar subregion may serve as a target used for ECT in treating MDD. Therefore, our findings on brain regions associated with anxiety, depression, or both may inform the selection of optimal targets for brain stimulation therapies. Future studies should investigate the relationship between brain stimulation-induced functional reorganization and GSCORR alterations to provide novel insights into the mechanisms of MDD and its treatment.

Additionally, anxious MDD patients exhibited decreased fALFF at the whole-brain level compared to HC and increased ALFF in the right anterior cingulate cortex compared to nonanxious MDD patients, consistent with previous findings [9, 10]. In contrast, the GSCORR revealed regional alterations across multiple brain regions, including the insula, precentral gyrus, and posterior parieto-occipital cortex. These results support our initial hypothesis that GSCORR can identify more brain abnormalities than ALFF/fALFF, suggesting that alterations in GSCORR may effectively detect subtle brain disruptions in anxious MDD patients. This further confirms that using a global topographic dynamic approach to investigate disease-related changes may provide a more comprehensive neural context for specific regions, networks, and frequencies, compared to a localization perspective [18].

This study has several limitations. First, there is a lack of extensive physiological and pathological data. Since the GS is significantly affected by physiological noise and movement, future research could enhance our understanding by integrating neurobiological data to explore the pathological underpinnings of GS alterations. Second, detailed information regarding medication use in MDD patients was unclear. To mitigate the potential confounding effects of antidepressant medication on GS topography, it is crucial to replicate our findings in a drug-naive cohort. Third, the cross-sectional design of our study limits causal inferences; longitudinal studies would be more effective in assessing how GS topography abnormalities evolve with disease progression. Fourth, we did not define a minimum threshold score to categorize depressive symptom severity as mild, moderate, or severe. Given the potential influence of symptom severity on fMRI results, future studies should incorporate such classifications to better account for this variability. Fifth, studies using SFI in anxiety and depression are currently limited. Future research is necessary to validate our findings and explore the potential clinical applications of SFI. Finally, employing different neuroimaging techniques may provide complementary insights into GS changes. Future studies could utilize diffusion MRI to explore relationships between structural connectivity and altered GS.

## 5. Conclusion

In this study, we utilized the REST-meta-MDD database to investigate alterations in GS topography in anxious and nonanxious MDD, as well as their associations with clinical assessments. Our results revealed anxiety-related disruptions in GS topography across extensive brain regions, especially in the precentral gyrus, insula, and posterior parieto-occipital cortex. Moreover, we observed SFI decreases in the SEN and increases in the default mode network among anxious MDD patients. These findings offer a novel perspective on understanding brain dysfunction in anxious MDD through a global topographic approach.

## Figures and Tables

**Table 1:** Demographic data in the three groups.

Group	HC^a^ (*N* = 307)	MDD_nonAnxiety^b^ (*N* = 145)	MDD_Anxiety^c^ (*N* = 334)	*p*	Post hoc
Age	37.81 ± 14.54	31.01 ± 10.83	35.57 ± 12.08	<0.001	a >b, a >c, b <c
Gender (M:F)	122 : 185	62 : 83	110 : 224	0.07	—
Education (year)	11.81 ± 2.69	11.86 ± 3.02	11.34 ± 3.16	0.08	—
Duration (month)	—	42.18 ± 55.05	50.40 ± 74.24	0.02	—
Episode					
First episode	—	66 (45.52%)	207 (61.98%)	—	—
Recurrent	—	60 (41.38%)	81 (24.25%)	—	—
No recoding	—	19 (13.10%)	46 (13.77%)	—	—
Medication					
Drug use	—	16 (11.03%)	90 (26.95%)	—	—
Drug naïve	—	59 (20.68%)	129 (38.62%)	—	—
No recoding	—	70 (48.28%)	115 (34.43%)	—	—
Head motion (mm)	0.07 ± 0.04	0.06 ± 0.03	0.07 ± 0.04	0.08	—
HAMD	—	10.79 ± 8.80	21.37 ± 6.88	<0.001	—
HAMA	—	9.01 ± 3.69	23.53 ± 7.19	<0.001	—

Abbreviations: F, female; M, male.

^a^Healthy controls.

^b^Patients without anxiety.

^c^Patients with anxiety.

## Data Availability

The data that support the findings of this study are available from https://rfmri.org/maps.

## Supporting Information

Supporting Information Figure S1. GMV differences between groups at the network level and the regional level. Figure S2. Regional GSCORR and SFC alterations in anxious MDD compared with HC. Figure S3. Correlation between GSCORR and clinical assessments in nonanxious MDD group.
