# Supplementary material for: Altered Global Signal Topography in Major Depressive Disorder With and Without Anxiety
Source: Depress Anxiety. 2025 Jul 2;2025:3864020. doi: 10.1155/da/3864020 (PMC12240666; doi:10.1155/da/3864020)
Supplement: Supporting Information — Figure S1. GMV differences between groups at the network level and the regional level. Figure S2. Regional GSCORR and SFC alterations in anxious MDD compared with HC. Figure S3. Correlation between GSCORR and clinical assessments in nonanxious MDD group. [file 3864020.f1.docx]

**Supplementary Figures**


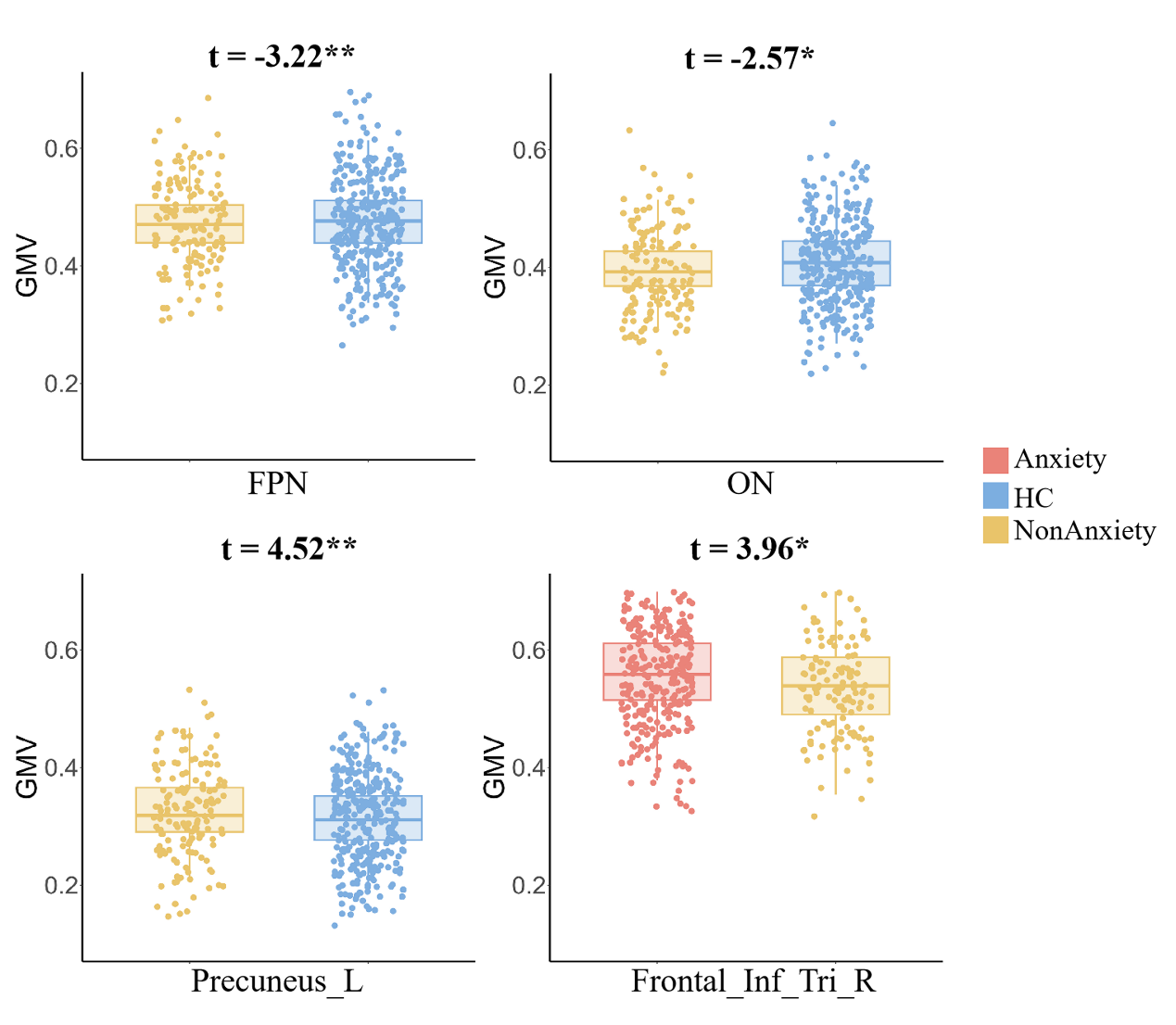


**Figure S1 GMV differences between groups at the network level (A) and the regional level (B).** Non-anxious MDD patients showed decreased GMV in frontoparietal and occipital networks, and showed increased GMV in left precuneus compared with HC. Furthermore, Anxious MDD showed increased GMV in right inferior frontal gyrus, triangular part compared with Non-anxious MDD. FPN: frontal parietal network; ON: occipital network. *:0.01< P <0.05, **:0.001 < P < 0.01.


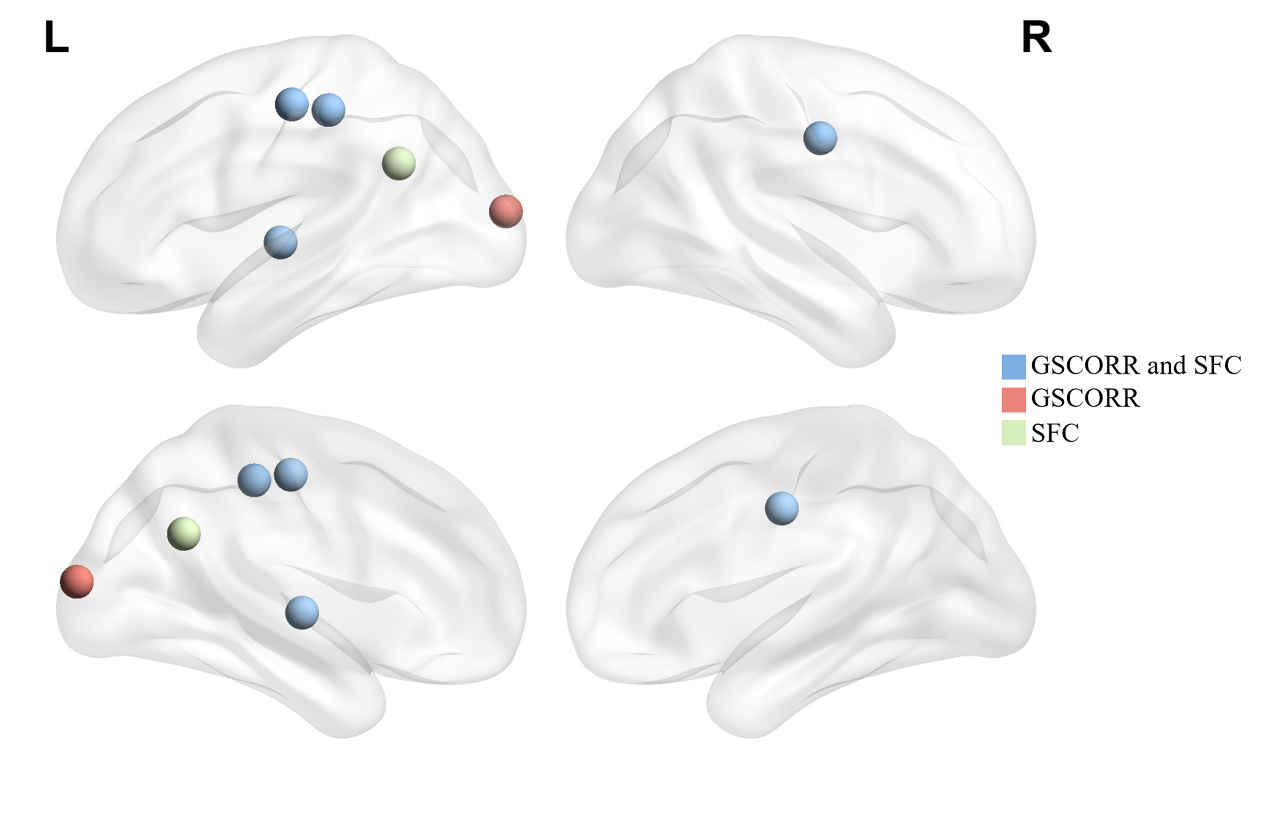


**Figure S2 Regional GSCORR and SFC alterations in anxious MDD compared with HC.** Regions that survived after FDR correction are shown. Node colors indicate the type of biomarker exhibiting significant alterations. The blue nodes represent regions with significant changes in both GSCORR and SFC. The red nodes indicate regions with significant changes in GSCORR only. The green nodes represent regions with significant alterations in SFC only.


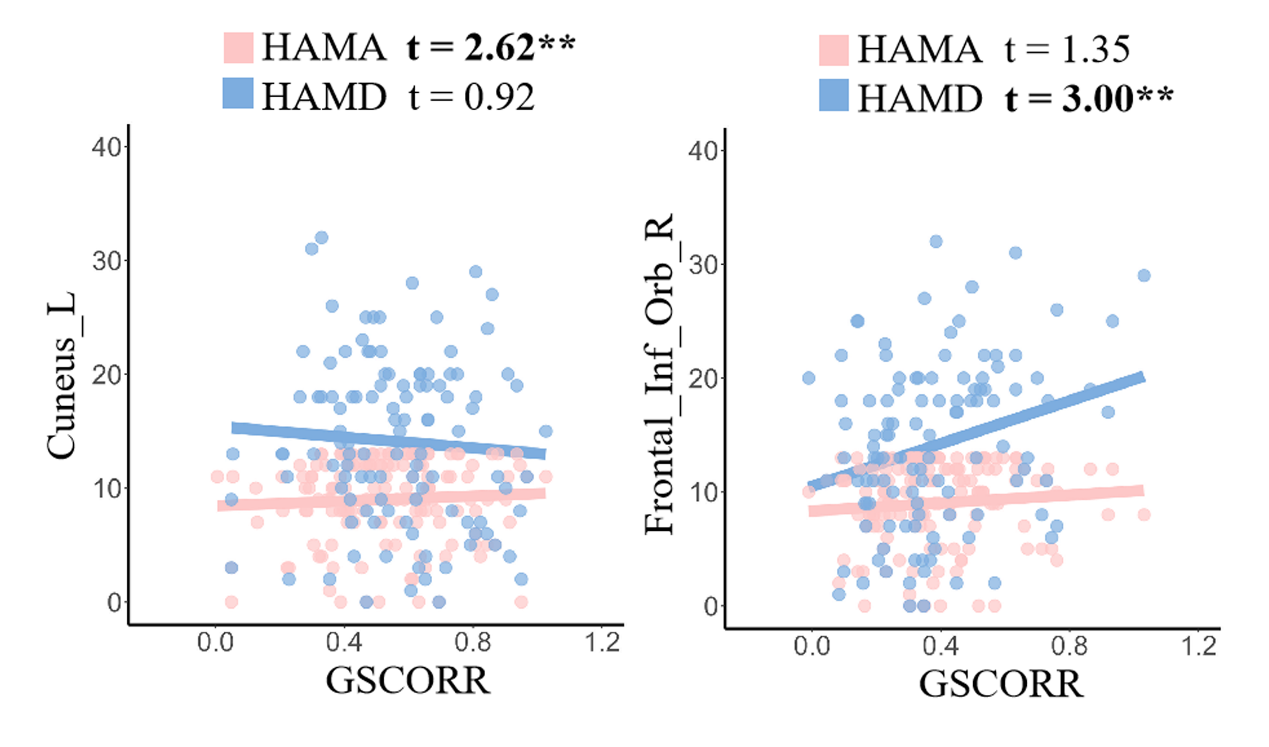


**Figure S3** **Correlation between GSCORR and clinical assessments in non-anxious MDD group.** Positive correlations between GSCORR in left cuneus and HAMA and between GSCORR in right inferior frontal gyrus and HAMD were observed. HAMD: Hamilton Depression Rating Scale, HAMA: Hamilton Anxiety Rating Scale.
